# Patient- and physician-reported outcomes from two phase 3 randomized studies (RAJ3 and RAJ4) of peficitinib (ASP015K) in Asian patients with rheumatoid arthritis

**DOI:** 10.1186/s13075-021-02590-z

**Published:** 2021-08-24

**Authors:** Yoshiya Tanaka, Tsutomu Takeuchi, Hiroyuki Izutsu, Yuichiro Kaneko, Daisuke Kato, Musashi Fukuda, Mitsuhiro Rokuda, Neil M. Schultz

**Affiliations:** 1 The First Department of Internal Medicine, School of Medicine, University of Occupational and Environmental Health, Japan, Kitakyushu, Japan; 2 Keio University School of Medicine, Tokyo, Japan; 3 Astellas Pharma Inc., Tokyo, Japan; 4 Astellas Pharma Global Development, Inc., Northbrook, Illinois USA

**Keywords:** Rheumatoid arthritis, Janus kinase (JAK), Peficitinib, ASP015K, Patient-reported outcomes (PROs), Work Productivity and Activity Impairment (WPAI) questionnaire, Health Assessment Questionnaire – Disability Index (HAQ-DI), Minimal clinically important difference (MCID)

## Abstract

**Background:**

Peficitinib (ASP015K), a novel oral Janus kinase inhibitor, has demonstrated efficacy and safety in the treatment of patients with rheumatoid arthritis (RA). This study evaluated the effect of peficitinib on patient- and physician-reported outcomes in Asian patients with RA and an inadequate response to prior disease-modifying antirheumatic drugs (DMARDs).

**Methods:**

Patients from two randomized, placebo-controlled, double-blind, phase 3 trials (RAJ3 and RAJ4) received once-daily peficitinib 100 mg, peficitinib 150 mg, or placebo, alone or in combination with DMARDs (RAJ3), or in combination with methotrexate (RAJ4). Mean changes in Work Productivity and Activity Impairment (WPAI) questionnaire domain scores from baseline, and percentages of patients achieving minimal clinically important differences (MCIDs) for patient- and physician-reported outcomes (WPAI, Health Assessment Questionnaire – Disability Index [HAQ-DI], and Subject’s Global Assessment of Pain [SGAP]), and Physician’s Global Assessment of disease activity (PGA) were evaluated at weeks 4, 8, 12, and 12/early termination (ET).

**Results:**

Data from 1025 patients were analyzed. At week 12/ET in both studies, patients who received peficitinib 100 mg or 150 mg reported significantly improved WPAI domain scores from baseline (except for absenteeism in RAJ4) compared with placebo (both doses, *p*<0.05). A higher proportion of peficitinib- versus placebo-treated patients achieved MCID in WPAI, HAQ-DI, SGAP, and PGA in studies RAJ3 and RAJ4. Significant differences with peficitinib versus placebo were evident in both studies as early as week 4 in HAQ-DI (peficitinib 150 mg only), SGAP, and PGA, and week 8 in WPAI loss of work productivity and daily activity impairment. At week 12/ET, significantly higher proportions of patients receiving peficitinib versus placebo achieved MCID in HAQ-DI, SGAP, PGA, and WPAI domains of presenteeism (RAJ3 only), loss of work productivity (RAJ3 only), and daily activity impairment (*p*<0.05 for all comparisons).

**Conclusions:**

Peficitinib 100 mg or 150 mg administered daily over 12 weeks resulted in clinically meaningful improvements in outcomes that are important to RA patients, including pain, physical function, and work productivity and activity. These observations were reinforced through similar improvements in physicians’ rating of disease activity.

**Trial registration:**

RAJ3: ClinicalTrials.gov, NCT02308163, registered 4 December 2014.

RAJ4: ClinicalTrials.gov, NCT02305849, registered 3 December 2014.

**Supplementary Information:**

The online version contains supplementary material available at 10.1186/s13075-021-02590-z.

## Background

Therapies for rheumatoid arthritis (RA) continue to advance, but nevertheless, the disability associated with RA can have a significant impact on patients’ physical and mental health, their social and working lives, sexual functioning, and consequently, their health-related quality of life [1–3].

To gain a comprehensive insight into the overall treatment effect of a therapy for RA, both physician-derived measures and patient-reported outcomes (PROs) should be assessed in clinical trials [4]. A range of PROs and physician-reported outcomes have been validated to evaluate health, quality of life, and treatment response in studies of patients with RA [4, 5]. These include a core set of PROs and physician-reported outcomes established by the American College of Rheumatology (ACR) [5, 6]. However, statistically significant changes in these outcome scores do not necessarily mean that the changes are clinically relevant [7, 8]. For this reason, interpretation of PRO and physician-reported data is improved by considering minimal clinically important differences (MCIDs), which help to determine the clinical relevance of observed changes, without relying solely on the presence or absence of statistical significance [7].

Peficitinib (ASP015K) is an oral, once-daily, pan-Janus kinase (JAK) inhibitor [9, 10], which is approved for use in Japan, Korea, and most recently, Taiwan, for the treatment of patients with RA who have an inadequate response to disease-modifying antirheumatic drugs (DMARDs) [11–13]. Two 52-week, randomized, controlled, phase 3 studies showed that once-daily peficitinib significantly reduced RA symptoms compared with placebo in Asian patients with RA and an inadequate response to DMARDs (RAJ3 study) or to methotrexate (MTX) specifically (RAJ4 study) [14, 15]. Peficitinib was well tolerated in these studies [14, 15]. Improvements versus placebo in PROs assessing pain, disability, and overall disease impact have been reported previously for peficitinib in patients with RA [14–16]. PROs have also been evaluated in phase 2 and 3 studies of other JAK inhibitors in patients with RA, and improvements compared to placebo have been reported for baricitinib, tofacitinib, upadacitinib, and filgotinib [17–24].

In the present analyses, we used data from both the RAJ3 and RAJ4 studies to assess the effect of peficitinib versus placebo on patient responses to the Work Productivity and Activity Impairment (WPAI) questionnaire. We also performed post hoc analyses of data from these studies to evaluate MCIDs for Physician’s Global Assessment of disease activity (PGA), and various PROs, including WPAI by employment status.

## Methods

### Study design and treatment

These analyses employed data from two 52-week, randomized, placebo-controlled, double-blind, parallel-group, phase 3, confirmatory studies of peficitinib for the treatment of RA, conducted at sites in Japan, Korea, and Taiwan (RAJ3; NCT02308163), or solely in Japan (RAJ4; NCT02305849) [14, 15]. Full details of these studies have been reported previously [14, 15].

Briefly, the RAJ3 and RAJ4 studies included patients with an inadequate response to DMARDs (RAJ3) or to MTX specifically (RAJ4) [14, 15]. Patients were randomized to receive once-daily oral placebo, or peficitinib 100 mg or 150 mg (alone or in combination with DMARDs [RAJ3], or in combination with MTX [RAJ4]) for 52 weeks [14, 15]. In the RAJ3 study, patients were also randomized to a once-weekly open-label etanercept (50 mg subcutaneous injection) group for use as a reference [14]. Patients (non-responders only in the RAJ4 study) who received placebo were switched at week 12 under blinded conditions to either peficitinib 100 or 150 mg, which was maintained until the end of treatment [14, 15]. All remaining RAJ4 patients who received placebo were switched to peficitinib at week 28 [15].

Key eligibility criteria for both studies have been published previously and included age ≥20 years, active RA (defined as ≥6/68 tender/painful joints and ≥6/66 swollen joints), and an inadequate response to, or intolerance of, at least one DMARD (RAJ3) or MTX (RAJ4) administered for ≥90 days prior to screening [14, 15].

### Physician- and patient-reported outcomes

In both studies, PROs and PGA were assessed at baseline and at weeks 4, 8, 12, and 12/early termination (ET) [14, 15]. PROs and PGA were also assessed at week 28/ET in RAJ4 [15]. As the period of comparison between peficitinib and placebo was restricted to the first 12 weeks in RAJ3 and RAJ4, PRO and PGA data beyond week 12 were not included in our analyses. Mean changes (with 95% confidence intervals) from baseline have been reported previously for Health Assessment Questionnaire – Disability Index (HAQ-DI), Subject’s Global Assessment of disease activity (SGA), Subject’s Global Assessment of Pain (SGAP), and PGA [14, 15].

Outcomes assessed for this secondary publication were mean changes from baseline in WPAI domain scores, with higher scores representing greater activity impairment and less productivity, and achievement of MCIDs (post hoc analyses) defined as decrease from baseline of ≥0.22 on a scale of 0–3 for HAQ-DI, ≥10 mm on a scale of 0–100 mm (visual analog scale) for SGAP and PGA, and ≥7% for WPAI domain scores [25–30]. WPAI outcomes were assessed using the WPAI questionnaire for RA, which included absenteeism (work time missed due to RA), presenteeism (impairment while working due to RA), loss of work productivity (overall work impairment due to RA), and daily activity impairment (activity impairment due to RA) domains [27, 31] and were expressed as percentage impairment. Mean changes from baseline and achievement of MCID in WPAI was further analyzed by employment status, defined as full-time paid worker (FTW; employed for ≥35 h/week), part-time paid worker (PTW; employed for <35 h/week), and homemaker (HM; unemployed, or employed in a capacity other than paid workers, and able to perform basic activities of daily life [household duties, shopping, child care, exercise, and study]), as detailed in Supplementary Methods [31]. Only daily activity impairment data were obtained for HMs.

### Statistical analysis

The analysis set included all patients who were randomized and received ≥1 dose of peficitinib, placebo, or reference treatment (etanercept) [14, 15]. The etanercept arm was an open-label reference group and was not included in statistical comparisons for efficacy endpoints [14]. Continuous variables were analyzed using analysis of covariance (ANCOVA). Between-treatment differences in achievement of MCIDs (peficitinib versus placebo) were evaluated in the RAJ3 study using the chi-squared test and in the RAJ4 study using Fisher’s exact test.

All time points included observed data only, except for week 12/ET. For week 12/ET, the last observation carried forward (LOCF) method was used for missing WPAI, HAQ-DI, SGAP, or PGA scores. Pearson correlation coefficients (r) were used to measure the strength of the relationship between change from baseline in WPAI domain scores (absenteeism, presenteeism, loss of work activity, and daily activity impairment) and clinical responses (28-joint disease activity score [DAS] based on C-reactive protein [CRP] and Clinical Disease Activity index [CDAI]) at week 12/ET post hoc.

## Results

### Patient populations

Overall, 1025 patients were included in the analysis set (507 in RAJ3 and 518 in RAJ4) [14, 15]. Patient disposition has been reported previously [14, 15]. Demographic characteristics, baseline disease activity, and RA history were generally similar across the treatment groups in the RAJ3 and RAJ4 studies [14, 15]. Mean RA duration was 8.89 years in the RAJ3 study and 4.36 years in the RAJ4 study, which reflected the different inclusion criteria for RA duration in these two study populations [14, 15]. In the RAJ3 study, there were no limitations for the duration of RA, but in the RAJ4 study, there was a limit of <10 years [14, 15].

In the RAJ3 study, TEAEs were similar across peficitinib 100 mg (56.7%), peficitinib 150 mg (53.9%), and placebo (53.5%) groups at week 12 [14]. In the RAJ4 study, TEAEs were reported in peficitinib 100 mg (51.1%), peficitinib 150 mg (59.8%), and placebo (49.4%) groups at week 12 [15].

Around half of patients were employed at baseline in the RAJ3 study across peficitinib 100 mg (59.2% [61/103]), peficitinib 150 mg (52.0% [53/102]), and placebo (50.0% [50/100]) groups, and in the RAJ4 study across peficitinib 100 mg (48.3% [84/174]), peficitinib 150 mg (60.7% [105/173]) and placebo (61.5% [104/169]) groups.

WPAI domain scores were similar across treatment groups at baseline for both the RAJ3 and RAJ4 studies, but absenteeism for the placebo group was numerically lower in RAJ3 and higher in RAJ4 compared with the comparator groups (Tables 1 and 2).

**Table 1:** Baseline WPAI scores: RAJ3

WPAI domain^a^	Placebo (*N*=101)	Peficitinib 100 mg (*N*=104)	Peficitinib 150 mg (*N*=102)	Peficitinib 100 mg + 150 mg (*N*=206)	*p* value^b^
Absenteeism^c^					
*n*	50	60	53	113	0.385
Mean (SD)	4.29 (11.10)	7.61 (22.79)	9.52 (21.17)	8.50 (21.97)	
Presenteeism^c^					
*n*	50	57	53	110	0.639
Mean (SD)	47.00 (28.94)	41.58 (28.83)	44.53 (31.04)	43.00 (29.82)	
Loss of work productivity^c^					
*n*	50	57	53	110	0.529
Mean (SD)	48.89 (28.89)	42.57 (29.46)	47.19 (31.85)	44.80 (30.58)	
Daily activity impairment					
*n*	100	103	102	205	0.543
Mean (SD)	55.90 (28.92)	51.75 (28.26)	55.20 (28.97)	53.46 (28.60)	

*SD* Standard deviation, *WPAI* Work Productivity and Activity Impairment

^a^Higher WPAI scores indicate greater work/activity impairment, expressed as percentage

^b^*p* values calculated using analysis of covariance, with no adjustment made for multiplicity

^c^Only “full-time paid workers” and “part-time paid workers” were included (not “homemakers”)

**Table 2:** Baseline WPAI scores: RAJ4

WPAI domain^a^	Placebo (*N*=170)	Peficitinib 100 mg (*N*=174)	Peficitinib 150 mg (*N*=174)	Peficitinib 100 mg + 150 mg (*N*=348)	*p* value^b^
Absenteeism^c^					
*n*	103	82	101	183	0.180
Mean (SD)	9.18 (22.43)	4.75 (11.04)	5.89 (14.78)	5.38 (13.21)	
Presenteeism^c^					
*n*	99	83	101	184	0.994
Mean (SD)	43.43 (26.15)	43.01 (30.71)	43.37 (28.05)	43.21 (29.20)	
Loss of work productivity^c^					
*n*	99	82	101	183	0.962
Mean (SD)	45.28 (27.03)	44.37 (30.96)	45.53 (29.13)	45.01 (29.88)	
Daily activity impairment					
*n*	169	173	173	346	0.471
Mean (SD)	52.60 (29.18)	48.84 (29.33)	50.69 (26.36)	49.77 (27.86)	

*SD* Standard deviation, *WPAI* Work Productivity and Activity Impairment

^a^Higher WPAI scores indicate greater work/activity impairment, expressed as percentage

^b^*p* values calculated using analysis of covariance, with no adjustment made for multiplicity

^c^Only “full-time paid workers” and “part-time paid workers” were included (not “homemakers”)

### Changes from baseline

#### Work productivity and activity impairment

**Fig. 1:**
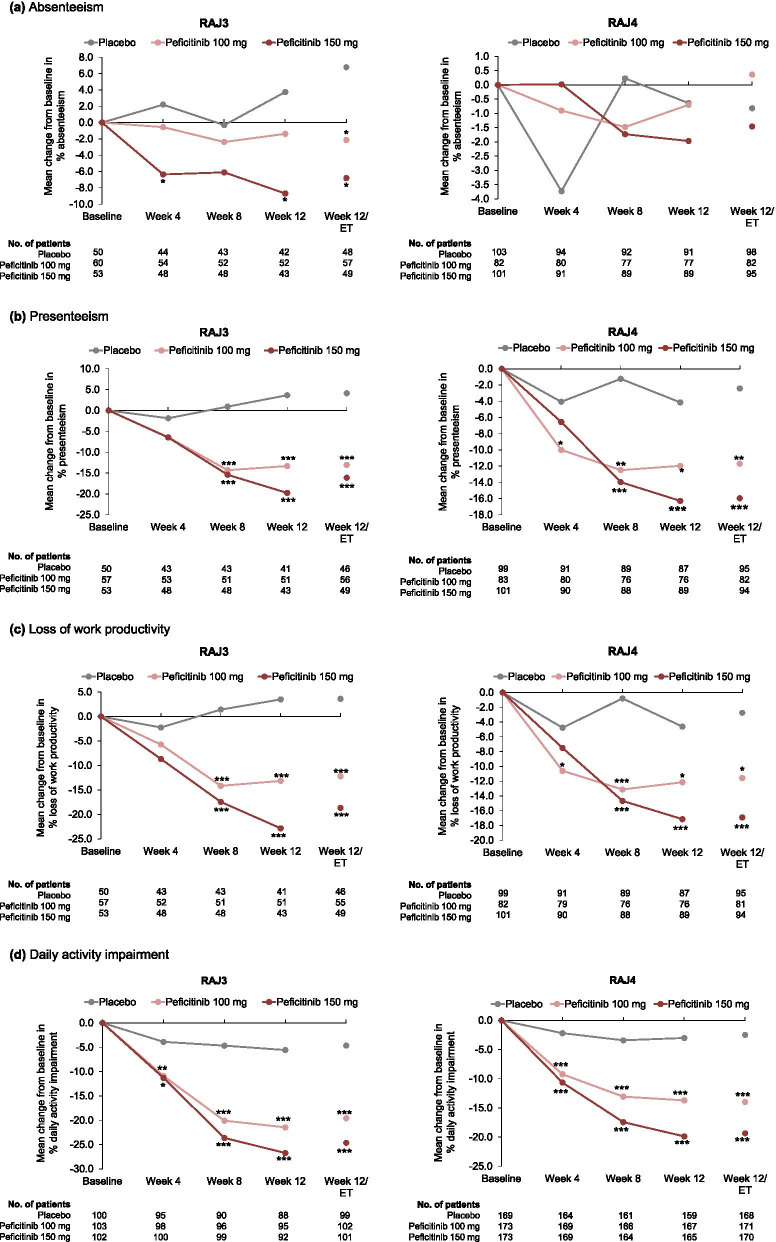
Mean changes from baseline to week 12/ET for WPAI domains. Except for “daily activity impairment,” only full-time paid workers and part-time paid workers were included (not homemakers). Higher WPAI scores indicate greater activity impairment and less productivity. WPAI outcomes are expressed as impairment percentages. All time points except for week 12/ET are observed data. At week 12/ET, last observation carried forward was used for missing WPAI scores. *p* values calculated using analysis of covariance, with no adjustment made for multiplicity. *p<0.05; **p<0.01; ***p<0.001, peficitinib versus placebo. *ET* early termination, *WPAI* Work Productivity and Activity Impairment

Clear trends for improvement in all WPAI domains were evident with both peficitinib 100 mg and 150 mg in the RAJ3 study, except for absenteeism with peficitinib 100 mg. Mean changes from baseline at week 12/ET in percentage impairment for the four domains (values shown in the order peficitinib 100 mg, peficitinib 150 mg, placebo) were absenteeism −2.14, −6.80, 6.78; presenteeism −13.04, −16.12, 4.13; loss of work productivity −12.20, −18.68, 3.62; and daily activity impairment −19.61, −24.65, −4.65. Observations at weeks 4, 8, and 12 showed improved scores for peficitinib 100 mg and 150 mg versus placebo. At week 12/ET, statistically significant improvements were observed for the mean change from baseline in all WPAI domain scores with both peficitinib doses compared with placebo (*p*<0.05 for all) (Fig. 1a–d). Peficitinib 150 mg showed numerically greater improvements in WPAI domain scores compared with peficitinib 100 mg.

**Fig. 2:**
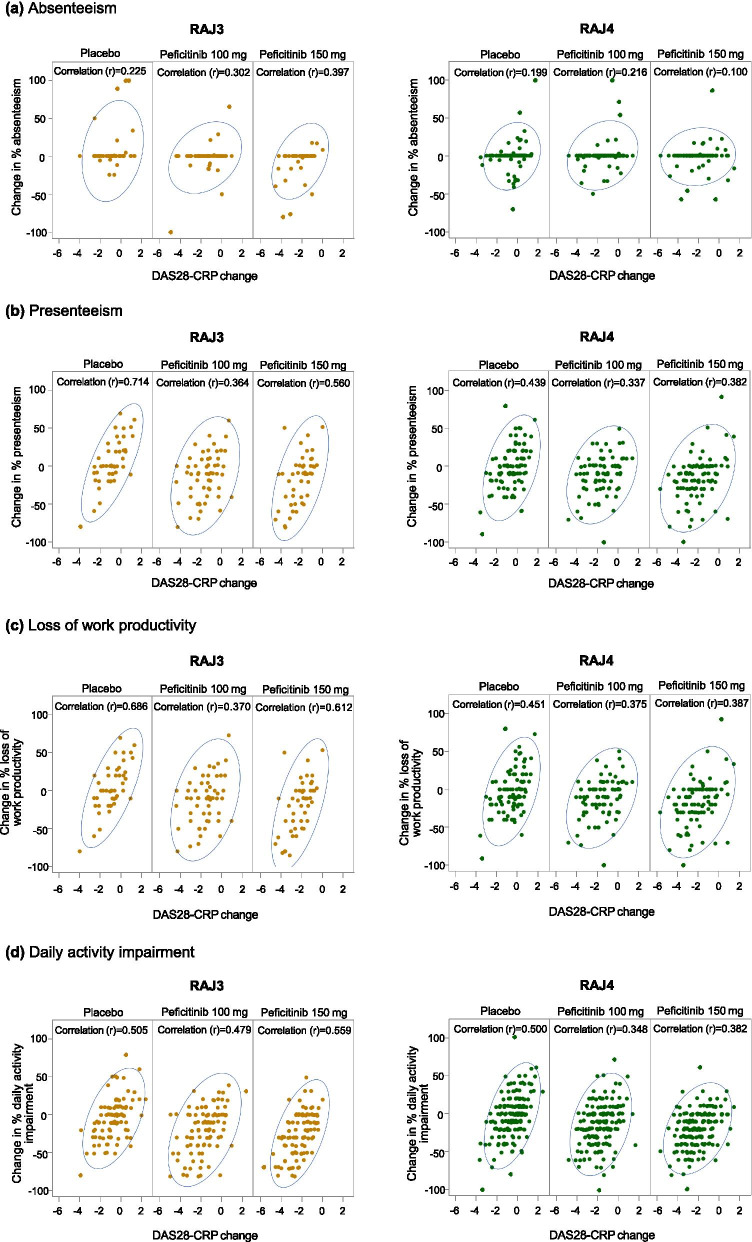
Correlations between WPAI domain scores and DAS28-CRP at week 12/ET. Except for “daily activity impairment,” only full-time paid workers and part-time paid workers were included (not homemakers). At week 12/ET, last observation carried forward was used for missing WPAI scores. The Pearson (*r*) correlation coefficients assessed the relationships between WPAI domains (absenteeism, presenteeism, loss of work productivity, and daily impairment) and clinical response (DAS28-CRP). A 95% prediction ellipse is displayed on each plot. DAS28-CRP, 28-joint disease activity score based on C-reactive protein. *ET* early termination, *WPAI* Work Productivity and Activity Impairment

There were moderate trends for positive correlations between WPAI change from baseline and clinical efficacy (DAS28-CRP and CDAI) for all WPAI domains, except absenteeism in RAJ3 (*r*=0.309–0.714 among all treatment groups; Fig. 2a–d and Supplementary Figure 1a–d).

In the RAJ4 study, both peficitinib 100 and 150 mg were associated with significant improvements from baseline to week 12/ET compared with placebo in three of the four WPAI domains (presenteeism, loss of work productivity, and daily activity impairment; *p*<0.05 for all three domains; Fig. 1a–d). Mean changes from baseline to week 12/ET in percentage impairment for the four domains (values shown in the order peficitinib 100 mg, peficitinib 150 mg, placebo) were absenteeism 0.36, −1.46, and −0.82; presenteeism −11.71, −15.96, and −2.42; loss of work productivity −11.58, −16.91, and −2.75; and daily activity impairment −13.98, −19.35, and −2.50. Observations at weeks 4, 8, and 12 showed improved scores in the WPAI domains, except for absenteeism, for peficitinib 100 and 150 mg versus placebo. In general, peficitinib 150 mg showed numerically greater improvements in scores compared with peficitinib 100 mg. No trend for improvement in absenteeism was observed with either peficitinib 100 or 150 mg (Fig. 1a).

In line with the RAJ3 study, there were moderate trends for positive correlations between improvements in WPAI domain scores and reduced disease activity (DAS28-CRP and CDAI) at week 12/ET in RAJ4 for all WPAI domains, except absenteeism (*r*=0.337–0.500 among all treatment groups; Fig. 2a–d and Supplementary Figure 1a–d).

#### Work productivity and activity impairment by employment status

There were significant improvements with both peficitinib 100 and 150 mg versus placebo in three of the four WPAI domains (presenteeism, loss of work productivity, and daily activity impairment; *p*<0.01 for all) among FTWs and PTWs at week 12/ET in RAJ3 (Supplementary Figure 2a–d). No significant differences were observed for absenteeism in FTWs for either dose of peficitinib or in PTWs for peficitinib 100 mg versus placebo (Supplementary Figure 2a), although absenteeism was significantly reduced for peficitinib versus placebo in the overall analysis set. In RAJ4, outcomes in FTWs and PTWs were broadly similar to those for the overall analysis set at week 12/ET, but statistically significant differences versus placebo were not observed for presenteeism and loss of work productivity in FTWs receiving peficitinib 100 mg (Supplementary Figure 2a–d). In HMs, daily activity impairment was significantly improved with both peficitinib doses versus placebo in RAJ3 and RAJ4 (*p*<0.05 for all; Supplementary Figure 2d), with improvements broadly similar to those for the overall analysis set.

### Minimal clinically important differences

#### Work productivity and activity impairment

**Fig. 3:**
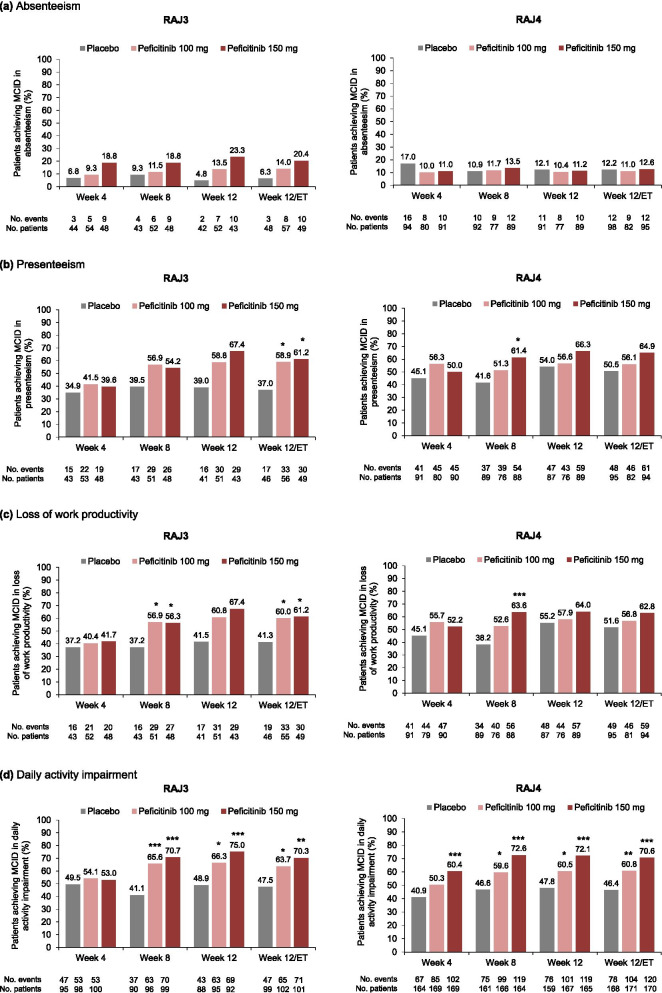
Proportion of patients achieving MCID in WPAI through week 12/ET. MCID for WPAI was defined as ≥7% decrease from baseline. Except for “daily activity impairment,” only full-time paid workers and part-time paid workers were included (not homemakers). All time points except for week 12/ET are observed data. At week 12/ET, last observation carried forward was used for missing scores. *p* values calculated using the chi-squared test (RAJ3) or Fisher’s exact test (RAJ4), with no adjustment made for multiplicity. *p<0.05; **p<0.01; ***p<0.001, peficitinib versus placebo. *p* value not estimable (RAJ3 only) in absenteeism at any time point, or presenteeism and loss of work productivity for peficitinib 150 mg at week 12. *ET* early termination, *MCID* minimal clinically important difference, *WPAI* Work Productivity and Activity Impairment

In the RAJ3 study, a significantly greater proportion of peficitinib-treated patients achieved MCID in three of the four WPAI domains at week 12/ET (presenteeism, loss of work productivity, and daily activity impairment), compared with placebo (*p*<0.05 for both doses and all three domains; Fig. 3a–d). The respective proportions of patients achieving MCID in the four domains at week 12/ET with peficitinib 100 mg, peficitinib 150 mg, and placebo were absenteeism 14.0, 20.4, and 6.3%; presenteeism 58.9, 61.2, and 37.0%; loss of work productivity 60.0, 61.2, and 41.3%; and daily activity impairment 63.7, 70.3, and 47.5% (Fig. 3a–d). The proportions of patients treated with peficitinib 100 or 150 mg achieving MCID were significantly higher versus placebo as early as week 8 for loss of work productivity and daily activity impairment (*p*<0.05 for both domains; Fig. 3c, d). A numerically greater proportion of patients receiving peficitinib 100 or 150 mg versus placebo achieved MCID at weeks 4, 8, 12, and 12/ET for the WPAI domain absenteeism (Fig. 3a).

In the RAJ4 study, a significantly higher proportion of patients treated with peficitinib 100 or 150 mg achieved MCID compared with placebo in one of the WPAI domains, daily activity impairment, from week 4 (peficitinib 150 mg) or week 8 (peficitinib 100 mg) to week 12/ET (*p*<0.05 for all time points; Fig. 3d). There were no significant differences found in the proportions of patients achieving MCID at week 12/ET in the other WPAI domains (Fig. 3a–c). The respective proportions of patients achieving MCID in the four domains at week 12/ET with peficitinib 100 mg, peficitinib 150 mg, and placebo were absenteeism 11.0, 12.6, and 12.2%; presenteeism 56.1, 64.9, and 50.5%; loss of work productivity 56.8, 62.8, and 51.6%; and daily activity impairment 60.8, 70.6, and 46.4% (Fig. 3a–d).

#### Work productivity and activity impairment by employment status

In RAJ3, the observations in FTWs (all four domains) and HMs (daily activity impairment only) appeared broadly similar to those for the overall analysis set (Supplementary Figure 3a–d). Among PTWs in RAJ3, there were no statistically significant differences between peficitinib and placebo in the proportions of patients achieving MCID in the four WPAI domains at week 12/ET (Supplementary Figure 3a–d). In RAJ4, the observations in FTWs and PTWs were broadly similar to those for the overall analysis set, but statistically significant differences versus placebo were not observed with peficitinib 100 mg for daily activity impairment at week 12/ET (Supplementary Figure 3a–d). Observations for daily activity impairment in HMs in RAJ4 reflected those for the overall analysis set (Supplementary Figure 3d).

#### Health Assessment Questionnaire – Disability Index

**Fig. 4:**
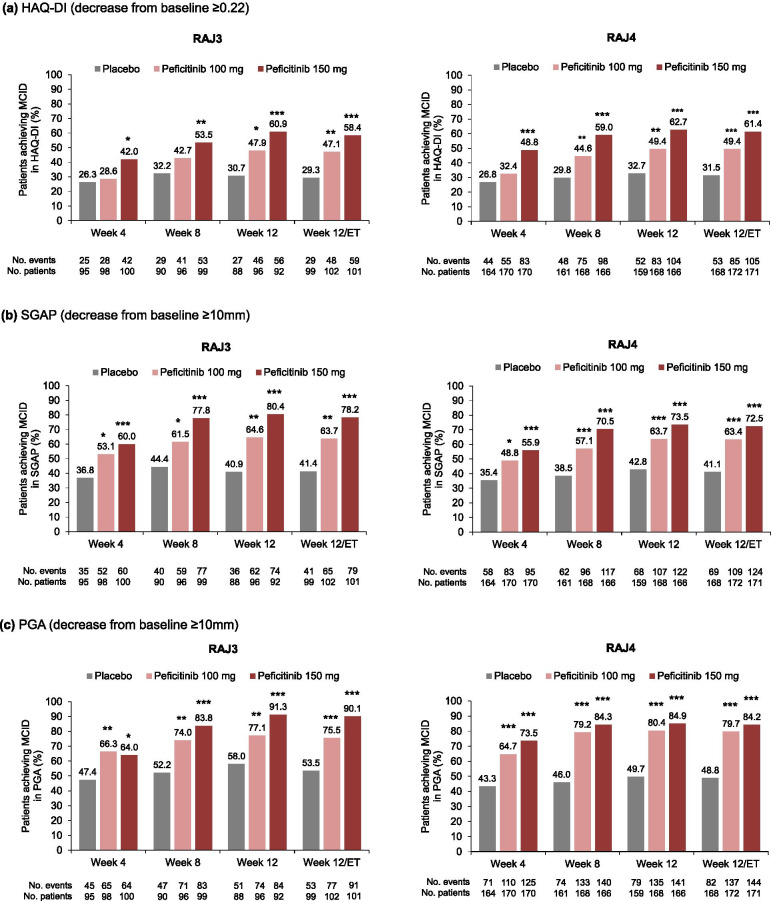
Proportion of patients achieving MCID in HAQ-DI, SGAP, and PGA through week 12/ET. MCID for HAQ-DI was defined as a decrease from baseline ≥0.22, SGAP as a decrease from baseline ≥10mm, and PGA as a decrease from baseline ≥10mm. All time points except for week 12/ET are observed data. At week 12/ET, last observation carried forward was used for missing scores. *p* values calculated using the chi-squared test (RAJ3) or Fisher’s exact test (RAJ4), with no adjustment made for multiplicity. *p<0.05; **p<0.01; ***p<0.001, peficitinib versus placebo. *ET* early termination, *HAQ-DI* Health Assessment Questionnaire – Disability Index, *MCID* minimal clinically important difference, *PGA* Physician’s Global Assessment of Arthritis, *SGAP* Subject’s Global Assessment of Pain

At week 12/ET, the proportion of patients achieving MCID in HAQ-DI was significantly greater with both peficitinib 100 mg and 150 mg versus placebo in the RAJ3 study (47.1% [*p*<0.01] and 58.4% [*p*<0.001] versus 29.3%, respectively) and the RAJ4 study (49.4 and 61.4% [*p*<0.001 for both doses] versus 31.5%, respectively; Fig. 4a).

A significantly greater proportion of patients receiving peficitinib 150 mg versus placebo achieved MCID in HAQ-DI from week 4 in both RAJ3 and RAJ4 (*p*<0.05 for both studies; Fig. 4a). In both studies, peficitinib 150 mg showed numerically greater achievement of MCID in HAQ-DI compared with peficitinib 100 mg (Fig. 4a).

#### Subject’s Global Assessment of Pain

At week 12/ET, the proportion of patients achieving MCID in SGAP was significantly greater with both peficitinib 100 mg and 150 mg versus placebo in the RAJ3 study (63.7% [*p*<0.01] and 78.2% [*p*<0.001] versus 41.4%, respectively) and the RAJ4 study (63.4 and 72.5% [*p*<0.001 for both doses] versus 41.1%, respectively; Fig. 4b). In both studies, significant differences versus placebo were observed for both peficitinib doses from week 4. Peficitinib 150 mg showed numerically greater achievement of MCID in SGAP compared with peficitinib 100 mg in both the RAJ3 and RAJ4 studies (Fig. 4b).

#### Physician’s Global Assessment of disease activity

At week 12/ET, the proportion of patients achieving MCID in PGA was significantly greater with both peficitinib 100 and 150 mg versus placebo in the RAJ3 study (75.5% [*p*<0.001] and 90.1% [*p*<0.001], respectively, versus 53.5%) and the RAJ4 study (79.7 and 84.2% [*p*<0.001 for both doses] versus 48.8%, respectively; Fig. 4c). In both studies, significant differences versus placebo were observed for both peficitinib doses from week 4. Peficitinib 150 mg showed numerically greater achievement of MCID in PGA compared with peficitinib 100 mg at all time points except week 4 in the RAJ3 study and at all time points in the RAJ4 study (Fig. 4c).

## Discussion

In RAJ3 and RAJ4, peficitinib 100 or 150 mg daily was efficacious in reducing RA symptoms and suppressing joint destruction and was well tolerated in patients with inadequate response to DMARDs (RAJ3) or MTX (RAJ4) [14, 15]. Significant improvements from baseline with peficitinib versus placebo were reported as early as week 4 for HAQ-DI, SGA, SGAP, and PGA [14, 15]. Statistical significance was maintained among the PROs and PGA through to week 12/ET (*p*<0.001 for all comparisons) [14, 15]. At week 12/ET, both peficitinib 100 and 150 mg were associated with significantly greater mean changes from baseline compared with placebo in scores for all four WPAI domains in the RAJ3 study and for three domains in the RAJ4 study. Among FTWs and PTWs at week 12/ET in the RAJ3 and RAJ4 studies, statistically significant improvements were observed in the mean change from baseline for peficitinib 100 and 150 mg compared to placebo across most WPAI domains. Clinically meaningful improvements in WPAI (with the exception of absenteeism), HAQ-DI, SGAP, and PGA were observed over 12 weeks in both studies, with most improvements evident as early as week 4. Also, we calculated positive trends for correlations between WPAI and the clinical efficacy of peficitinib. These findings demonstrate that peficitinib is associated with significant improvements in patient-reported measures of pain, disability, and activity; all are important indicators of the general quality of life and the patient’s ability to function.

In the RAJ3 and RAJ4 studies, only around half of the enrolled patients were employed, and patient numbers were therefore low for the absenteeism, presenteeism, and loss of work productivity analyses. This may have contributed to the lack of significant differences observed between the peficitinib and placebo groups in the absenteeism domain. In addition, the baseline absenteeism score was numerically higher in the RAJ4 placebo group compared with the peficitinib groups. Similar results to ours have been observed in absenteeism in work productivity analyses of other JAK inhibitors at week 12 [32–34]. Contrarily, clear dose-dependent and significant differences were observed in our study between the peficitinib and placebo groups in daily activity impairment, a domain which included FTWs, PTWs, and HMs and was therefore not affected by patients’ employment status. These positive changes in work productivity were likely related to clinical improvement with peficitinib. Of note, the proportion of patients achieving MCID in the placebo group was numerically higher in RAJ4 than in RAJ3 at all time points for absenteeism, presenteeism, and loss of work productivity, which was perhaps due to a numerically shorter duration of RA in RAJ4 than RAJ3.

The improvements in work productivity and activity observed with peficitinib versus placebo in our analyses are similar to those for other JAK inhibitors (baricitinib and upadacitinib) in global studies that included Asian patients with RA [17–19]. In a phase 3 study of baricitinib- or adalimumab-treated RA patients with an inadequate response to MTX (*N*=1305), significant improvements for baricitinib versus placebo were observed across all WPAI domains at week 12 (*p*≤0.05) [17]. Similar observations have been reported from a phase 3 study of upadacitinib in MTX-naïve RA patients (*N*=945) [35]. Patients receiving upadacitinib 15 or 30 mg daily reported significant improvements from baseline in daily activity impairment and loss of work productivity at week 12 compared with patients receiving MTX alone (*p*<0.01), and significantly, more upadacitinib-treated patients achieved the MCID (*p*<0.05) [35]. Of note in our study, peficitinib compared with placebo showed early (from week 4) and marked improvement in daily activity impairment. Improvements of a similar magnitude for this WPAI component have been reported for baricitinib, upadacitinib, and filgotinib in patients with RA [33–37]. A study of work productivity for RA patients receiving the anti-IL-6 receptor antibody tocilizumab also showed responses of a similar magnitude to peficitinib for improvements in daily activity impairment [38].

Functional ability, as measured by achievement of MCID in HAQ-DI, was significantly improved for peficitinib 150 mg versus placebo from week 4 in both the RAJ3 and RAJ4 studies. Early improvement in functional ability has been reported previously for peficitinib. In a 12-week phase 2b study in Japan (RAJ1), once-daily peficitinib 100 and 150 mg were associated with significant improvements in HAQ-DI versus placebo from week 1 [16]. Our observations at week 12/ET are consistent with reports from studies of tofacitinib in RA patients. In a phase 2 study in Japanese patients with RA and an inadequate response to MTX (*N*=136), a significantly higher proportion of patients receiving tofacitinib (3 mg, 5 mg, or 10 mg twice-daily) combined with MTX achieved MCID in HAQ-DI, compared with placebo, at week 12 (*p*<0.05 for all three doses) [20]. The proportion of tofacitinib-treated patients achieving MCID in HAQ-DI was also significantly higher compared with placebo at 3 months in each of two global phase 3 studies of tofacitinib (5 mg or 10 mg twice-daily) [21, 22]. Specifically, these studies enrolled patients receiving MTX with an inadequate response to tumor necrosis factor inhibitors (*N*=399; *p*<0.05 for both doses versus placebo), or inadequate response to conventional or biological DMARDS (*N*=792; *p*<0.0001 for both doses versus placebo) [21, 22]. Furthermore, our results are in line with reports from two global phase 3 studies of baricitinib (2 mg or 4 mg daily; *N*=684) and upadacitinib (15 mg or 30 mg daily; *N*=661) in RA patients with an inadequate response to csDMARDs [19, 23]. In these studies, the proportions of patients achieving MCID in HAQ-DI with JAK inhibitor were significantly improved compared with placebo at week 12 (*p*<0.05 for both doses in both studies) [19, 23].

We observed consistent improvements in SGAP MCID achievement with peficitinib versus placebo in the RAJ3 and RAJ4 studies. Our findings are similar to reports of clinically meaningful improvements in patient-assessed pain versus placebo for tofacitinib at 3 months (*p*<0.0001 for both 5 mg and 10 mg twice-daily) [21, 22] and upadacitinib at 12 weeks (*p*<0.05 for both 15 mg and 30 mg daily) [19], in patients with RA and an inadequate response to DMARDs; however, these data do not appear to have been reported for baricitinib.

Our analyses have limitations when interpreting the results. Although the PRO and PGA findings are consistent with the efficacy results of the RAJ3 and RAJ4 studies, we did not investigate whether the PRO findings were correlated with the efficacy results at the patient level. In addition, the observation periods for RAJ3 and RAJ4 were shorter than for some of the other JAK inhibitor studies [17, 24]. Longer observations may enable further correlations between PROs and RA symptoms. Despite filling a critical gap, the RAJ3 and RAJ4 studies included Asian populations only. It is recognized that Asian populations may have a different conceptualization of physical and mental health compared with Western populations [39]. Thus, it is important to consider cultural differences when interpreting PRO data. In addition, Japanese PRO data were evaluated using MCID criteria established in a non-Japanese population. A further limitation of the RAJ3 and RAJ4 studies was, for ethical reasons, placebo treatment was of shorter duration than the peficitinib arms, making comparisons difficult.

## Conclusions

Our findings strengthen the previously reported clinical efficacy and PRO data for peficitinib in Asian RA patient populations [14–16]. Peficitinib 100 or 150 mg administered daily over 12 weeks resulted in clinically meaningful improvements in outcomes that are important to patients, including pain, physical function, and work productivity and activity. These observations were reflected in similar improvements in physicians’ ratings of disease activity. Further studies are needed to understand the longer-term effects of peficitinib treatment on PROs.

## Supplementary Information

**Additional file 1: Supplementary Methods. Supplementary Figure 1.** Correlations between WPAI domain scores and CDAI at Week 12/ET. Except for ‘daily activity impairment’, only full-time paid workers and part-time paid workers were included (not homemakers). At Week 12/ET, last observation carried forward was used for missing WPAI scores. The Pearson (r) correlation coefficients assessed the relationships between WPAI domains (absenteeism, presenteeism, loss of work productivity, and daily impairment) and clinical response (CDAI). A 95% prediction ellipse is displayed on each plot. CDAI, Clinical Disease Activity Index. *ET* early termination, *WPAI* Work Productivity and Activity Impairment.** Supplementary Figure 2. **Mean changes from baseline at Week 12/ET for WPAI domains by employment status. Full-time paid workers and part-time paid workers were employed for ≥35 or <35 hours/week, respectively. Homemakers were unemployed, or employed in a capacity other than paid workers, and able to perform basic activities of daily life. Only daily activity impairment data were obtained for homemakers. Higher WPAI scores indicate greater activity impairment and less productivity. At Week 12/ET, last observation carried forward was used for missing WPAI scores. p-values calculated using analysis of covariance, with no adjustment made for multiplicity. *p<0.05; **p<0.01; ***p<0.001, peficitinib versus placebo. *ET* early termination, *WPAI* Work Productivity and Activity Impairment.** Supplementary Figure 3. **Proportion of patients achieving MCID in WPAI at Week 12/ET by employment status. MCID for WPAI was defined as ≥7% decrease from baseline. Full-time paid workers and part-time paid workers were employed for ≥35 or <35 hours/week, respectively. Homemakers were unemployed, or employed in a capacity other than paid workers, and able to perform basic activities of daily life. Only daily activity impairment data were obtained for homemakers. All time points except for Week 12/ET are observed data. At Week 12/ET, last observation carried forward was used for missing WPAI scores. p-values calculated using the chi-squared test (RAJ3) or Fisher’s Exact test (RAJ4), with no adjustment made for multiplicity. *p<0.05; **p<0.01, peficitinib versus placebo. *ET* early termination, *MCID* minimal clinically important difference, *NE* not estimable, *WPAI* Work Productivity and Activity Impairment.

## Data Availability

Researchers may request access to anonymized participant level data, trial level data, and protocols from Astellas sponsored clinical trials at www.clinicalstudydatarequest.com. For the Astellas criteria on data sharing, see https://clinicalstudydatarequest.com/Study-Sponsors/Study-Sponsors-Astellas.aspx.

## Abbreviations

ACR: American College of Rheumatology
ANCOVA: Analysis of covariance
CDAI: Clinical Disease Activity index
csDMARDs: Conventional synthetic disease-modifying antirheumatic drugs
DAS28-CRP: 28-Joint disease activity score based on C-reactive protein
DMARDs: Disease-modifying antirheumatic drugs
ET: Early termination
FTW: Full-time paid worker
HAQ-DI: Health Assessment Questionnaire – Disability Index
HM: Homemaker
JAK: Janus kinase
LOCF: Last observation carried forward
MCID: Minimal clinically important difference
MTX: Methotrexate
PGA: Physician’s Global Assessment of disease activity
PRO: Patient-reported outcomes
PTW: Part-time paid worker
RA: Rheumatoid arthritis
RA-WIS: Work Instability Scale for Rheumatoid Arthritis
SD: Standard deviation
SGA: Subject’s Global Assessment of disease activity
SGAP: Subject’s Global Assessment of Pain
WPAI: Work Productivity and Activity Impairment Questionnaire
