# Supplementary material for: Patient- and physician-reported outcomes from two phase 3 randomized studies (RAJ3 and RAJ4) of peficitinib (ASP015K) in Asian patients with rheumatoid arthritis
Source: Arthritis Res Ther. 2021 Aug 24;23:221. doi: 10.1186/s13075-021-02590-z (PMC8383363; doi:10.1186/s13075-021-02590-z)

# Supplementary Methods

Absenteeism, presenteeism, loss of work productivity, and daily activity impairment were calculated or assessed as follows, based on methods reported by Takeuchi *et al* [1]. Absenteeism (%) was calculated as: (hours absent from work due to rheumatoid arthritis [RA]/(hours absent from work due to RA + hours actually worked) x 100. Missed days per year were calculated from WPAI/absenteeism. Presenteeism was calculated using a visual analog scale (VAS) as the percentage of reduction in productivity due to RA while working. Loss of work productivity (%) was calculated as: absenteeism + ([1 **−** absenteeism] x presenteeism) x 100. Daily activity impairment was assessed using a VAS as a percentage of impairment due to RA in regular daily activities not including paid work [1].

## Supplementary reference

1. Takeuchi T, Nakajima R, Komatsu S, Yamazaki K, Nakamura T, Agata N, et al. Impact of Adalimumab on Work Productivity and Activity Impairment in Japanese Patients with Rheumatoid Arthritis: Large-Scale, Prospective, Single-Cohort ANOUVEAU Study. Adv Ther. 2017;34(3):686–702.

# Supplementary Figures

**Supplementary Figure 1.** Correlations between WPAI domain scores and CDAI at Week 12/ET. Except for ‘daily activity impairment’, only full-time paid workers and part-time paid workers were included (not homemakers). At Week 12/ET, last observation carried forward was used for missing WPAI scores. The Pearson (r) correlation coefficients assessed the relationships between WPAI domains (absenteeism, presenteeism, loss of work productivity, and daily impairment) and clinical response (CDAI). A 95% prediction ellipse is displayed on each plot. CDAI, Clinical Disease Activity Index; ET, early termination; WPAI, Work Productivity and Activity Impairment.

**(a)** Absenteeism

**
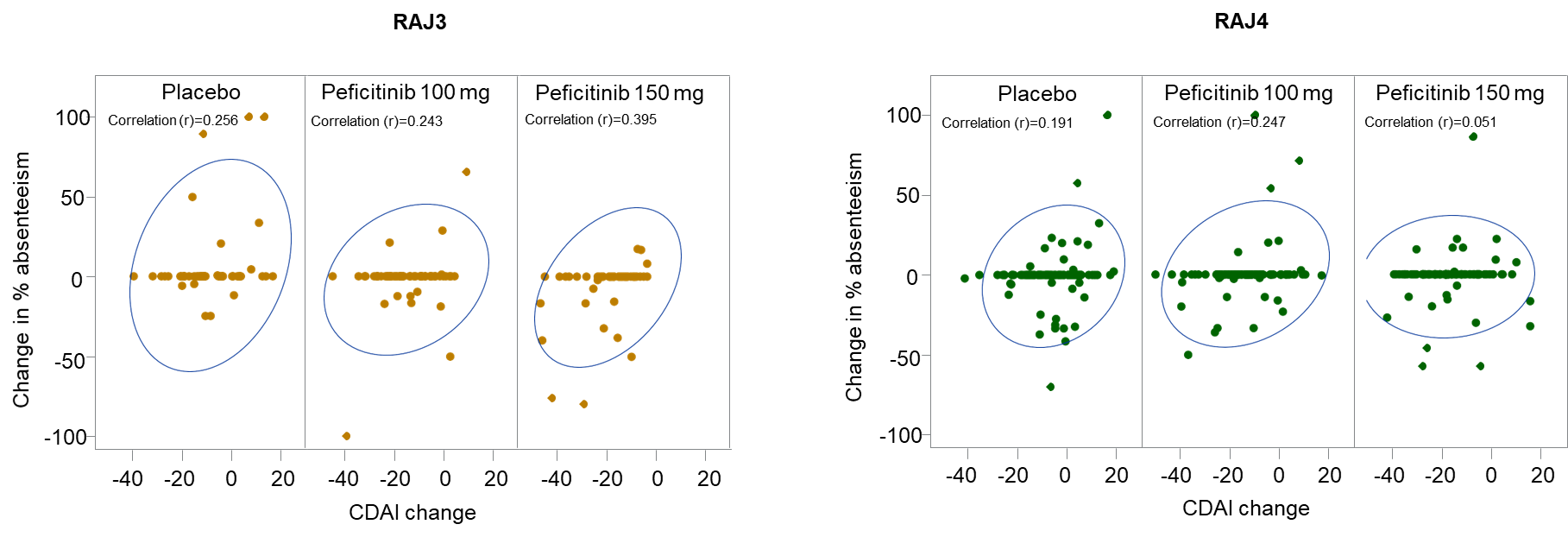
**

**(b)** Presenteeism


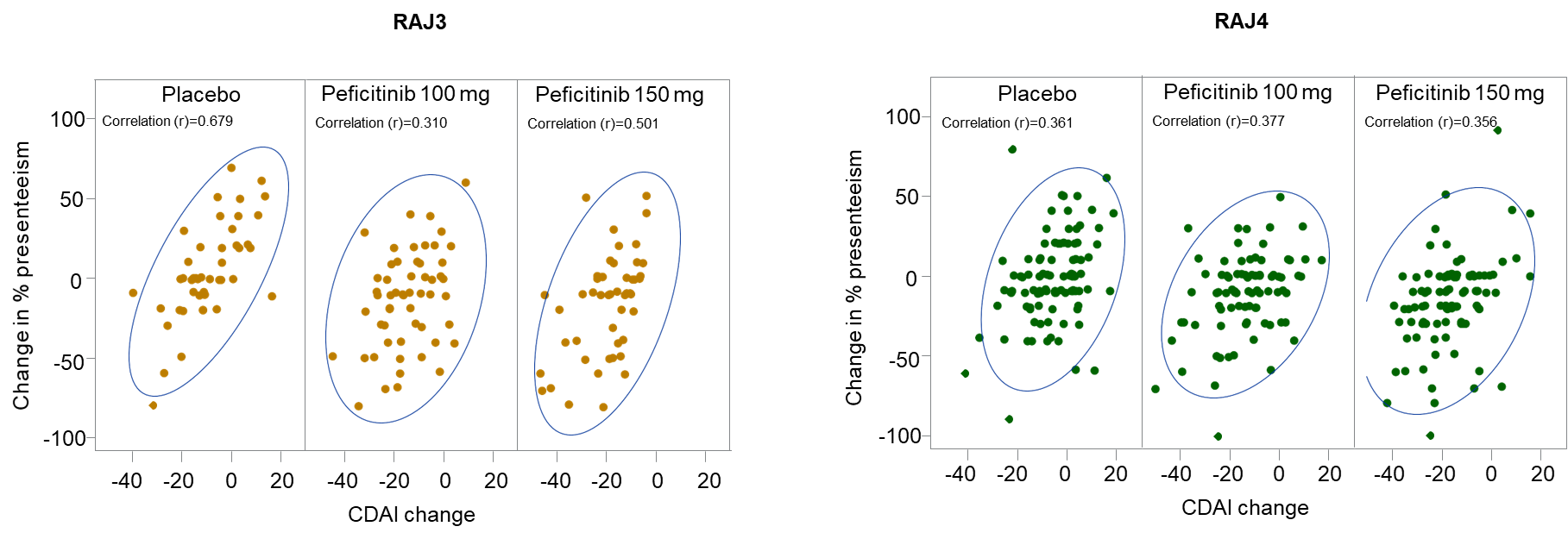


**(c)** Loss of work productivity


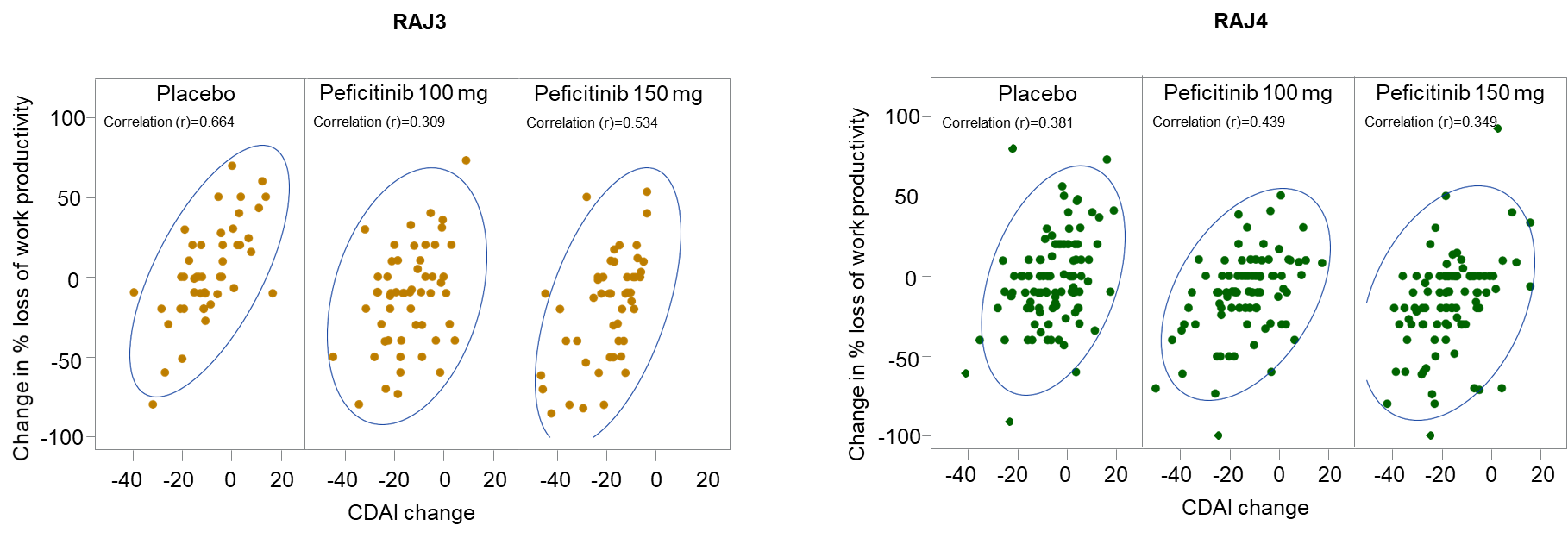


**(d)** Daily activity impairment


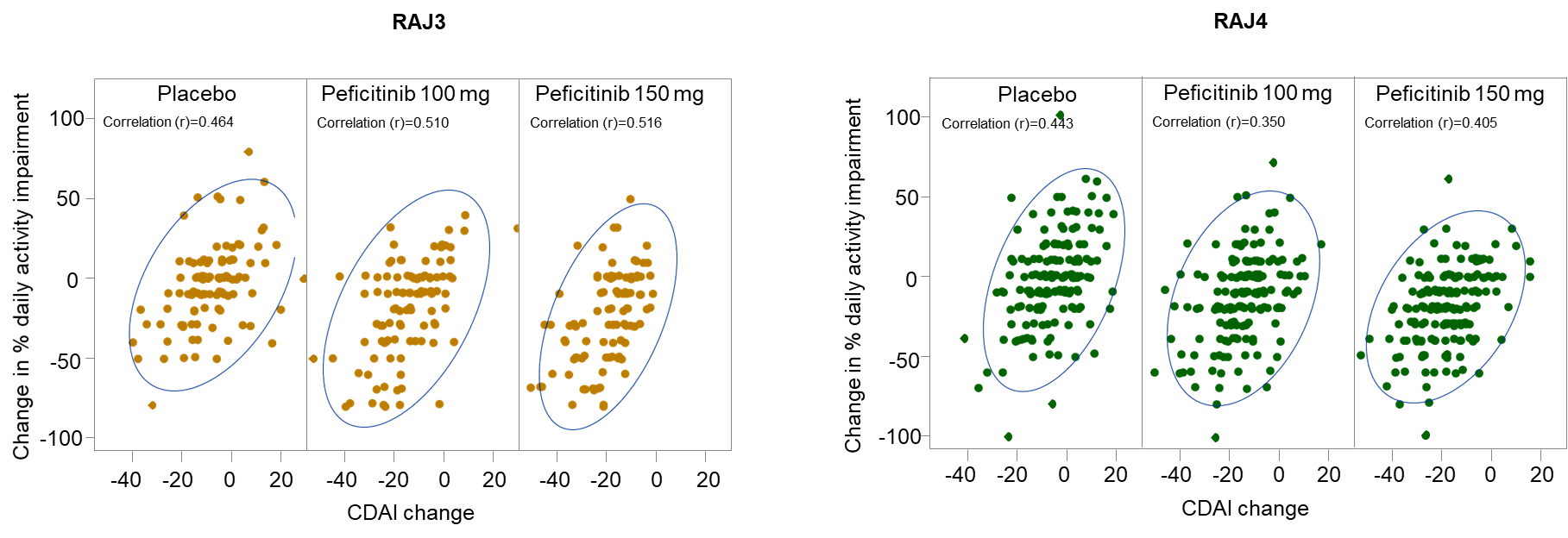


**Supplementary Figure 2.** Mean changes from baseline at Week 12/ET for WPAI domains by employment status. Full-time paid workers and part-time paid workers were employed for ≥35 or <35 hours/week, respectively. Homemakers were unemployed, or employed in a capacity other than paid workers, and able to perform basic activities of daily life. Only daily activity impairment data were obtained for homemakers. Higher WPAI scores indicate greater activity impairment and less productivity. At Week 12/ET, last observation carried forward was used for missing WPAI scores. p-values calculated using analysis of covariance, with no adjustment made for multiplicity. *p<0.05; **p<0.01; ***p<0.001, peficitinib versus placebo. ET, early termination; WPAI, Work Productivity and Activity Impairment.

**(a)** Absenteeism


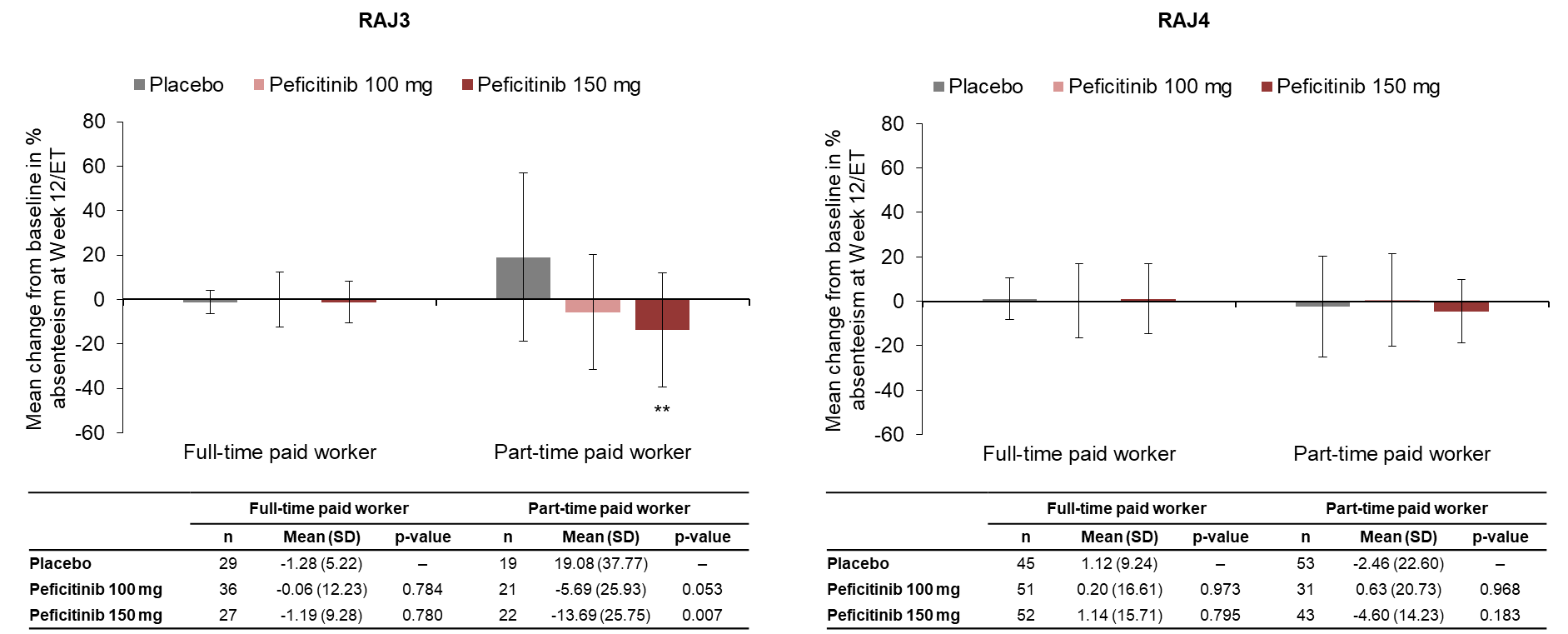


**(b)** Presenteeism


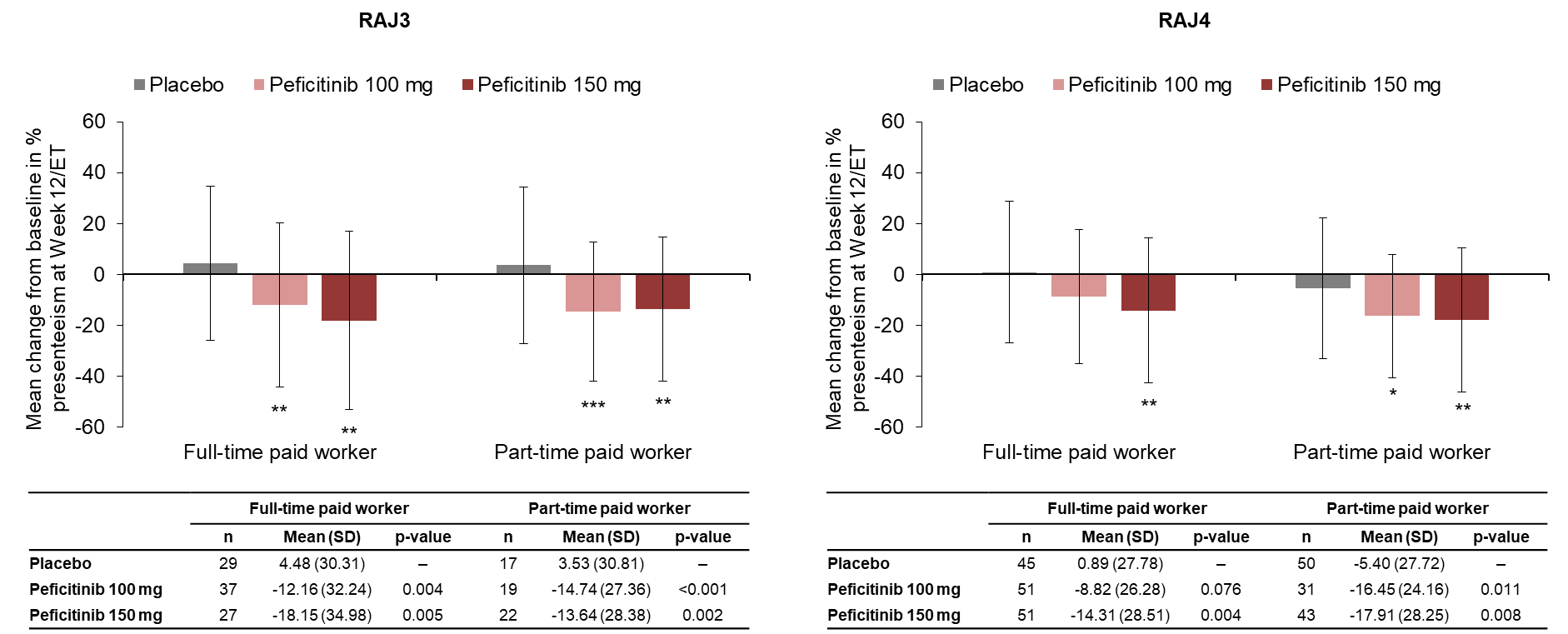


**(c)** Loss of work productivity


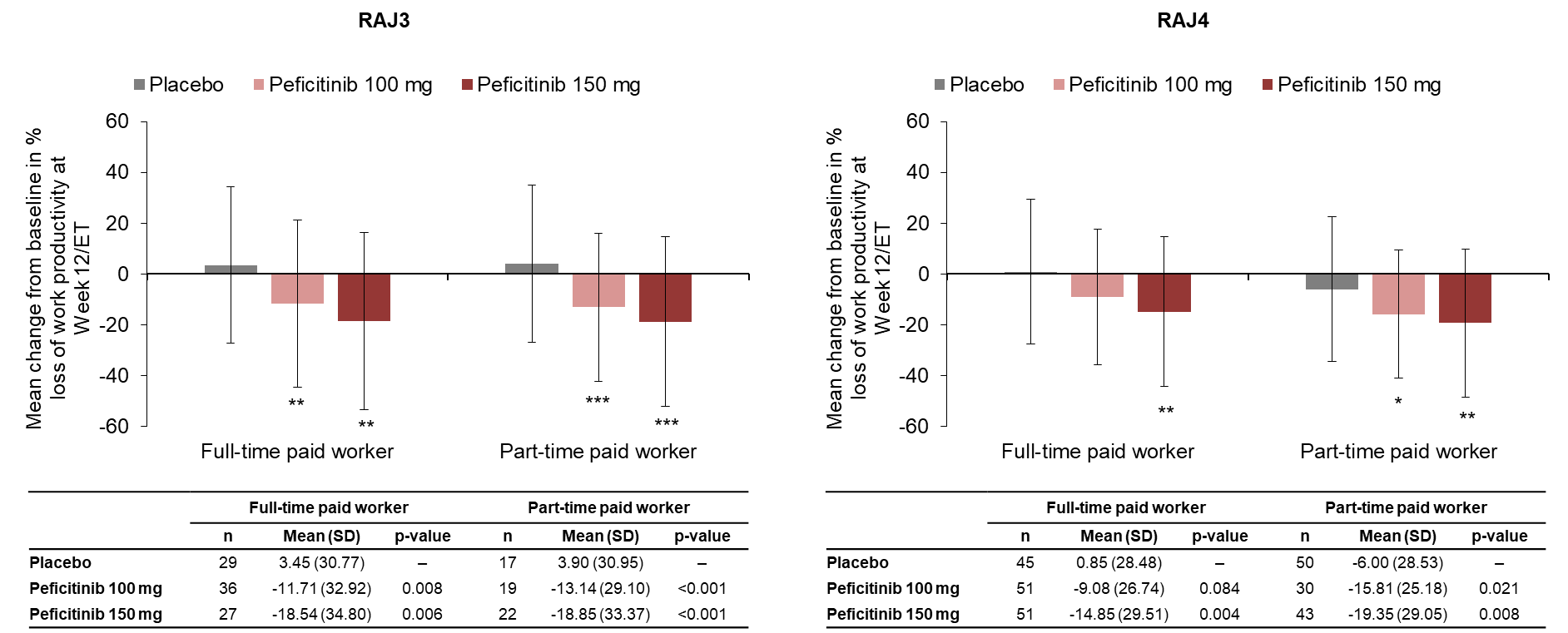


**(d)** Daily activity impairment


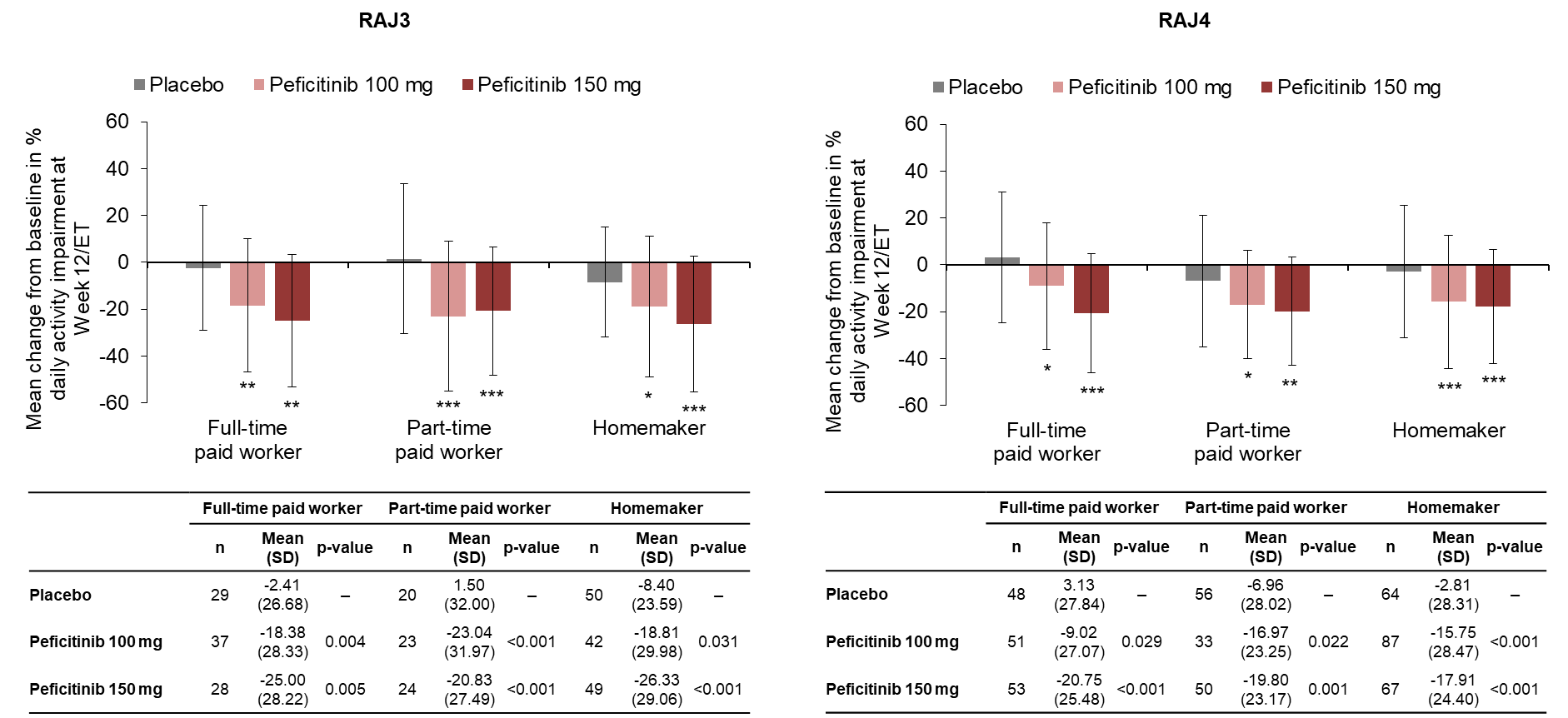


**Supplementary Figure 3.** Proportion of patients achieving MCID in WPAI at Week 12/ET by employment status. MCID for WPAI was defined as ≥7% decrease from baseline. Full-time paid workers and part-time paid workers were employed for ≥35 or <35 hours/week, respectively. Homemakers were unemployed, or employed in a capacity other than paid workers, and able to perform basic activities of daily life. Only daily activity impairment data were obtained for homemakers. All time points except for Week 12/ET are observed data. At Week 12/ET, last observation carried forward was used for missing WPAI scores. p-values calculated using the chi-squared test (RAJ3) or Fisher’s Exact test (RAJ4), with no adjustment made for multiplicity. *p<0.05; **p<0.01, peficitinib versus placebo. ET, early termination; MCID, minimal clinically important difference; NE, not estimable; WPAI, Work Productivity and Activity Impairment.

**(a)** Absenteeism


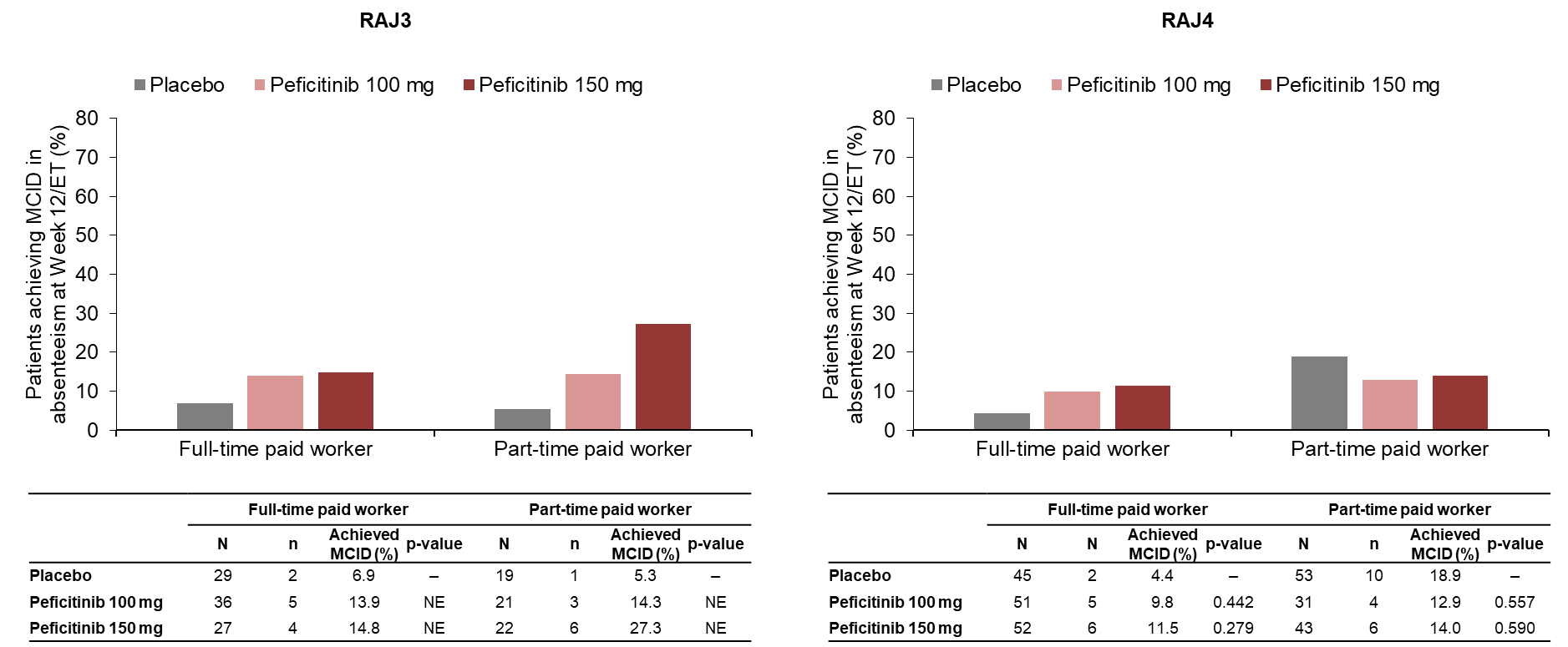


**(b)** Presenteeism


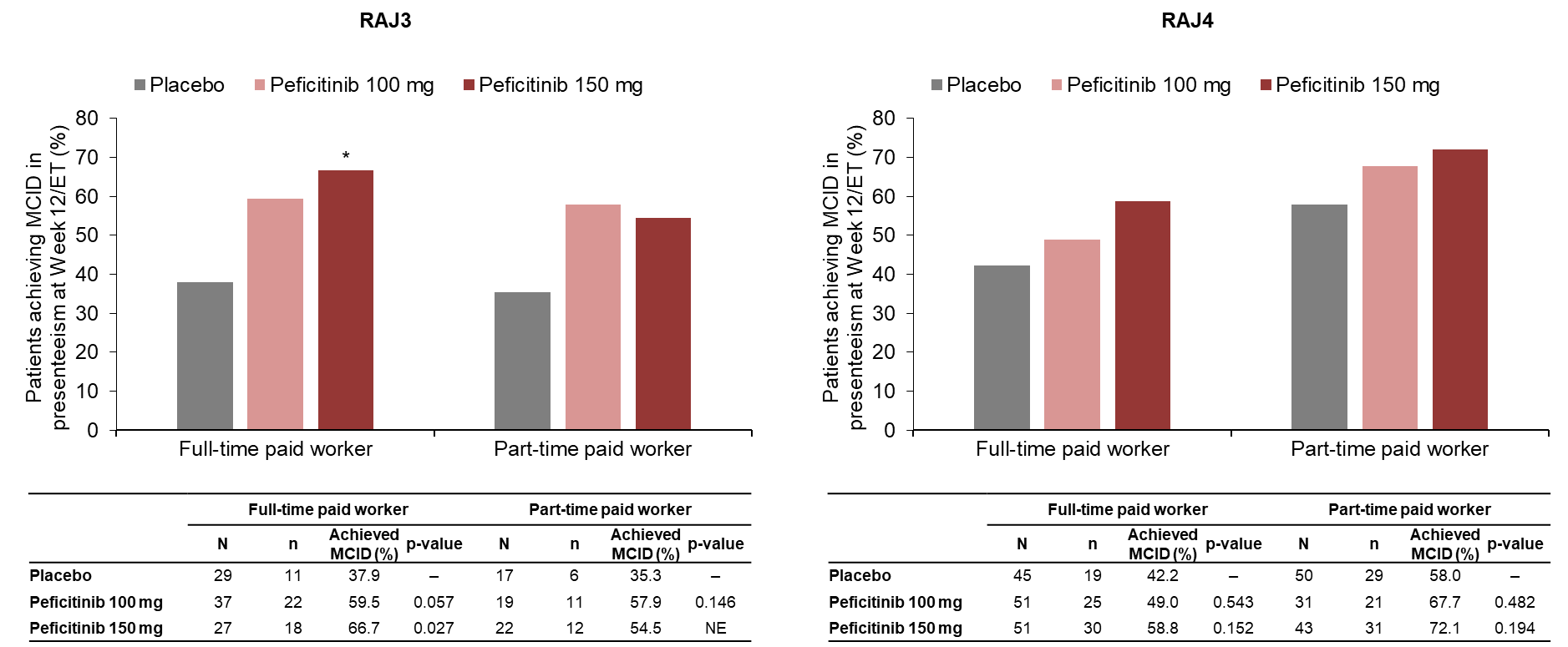


**(c)** Loss of work productivity


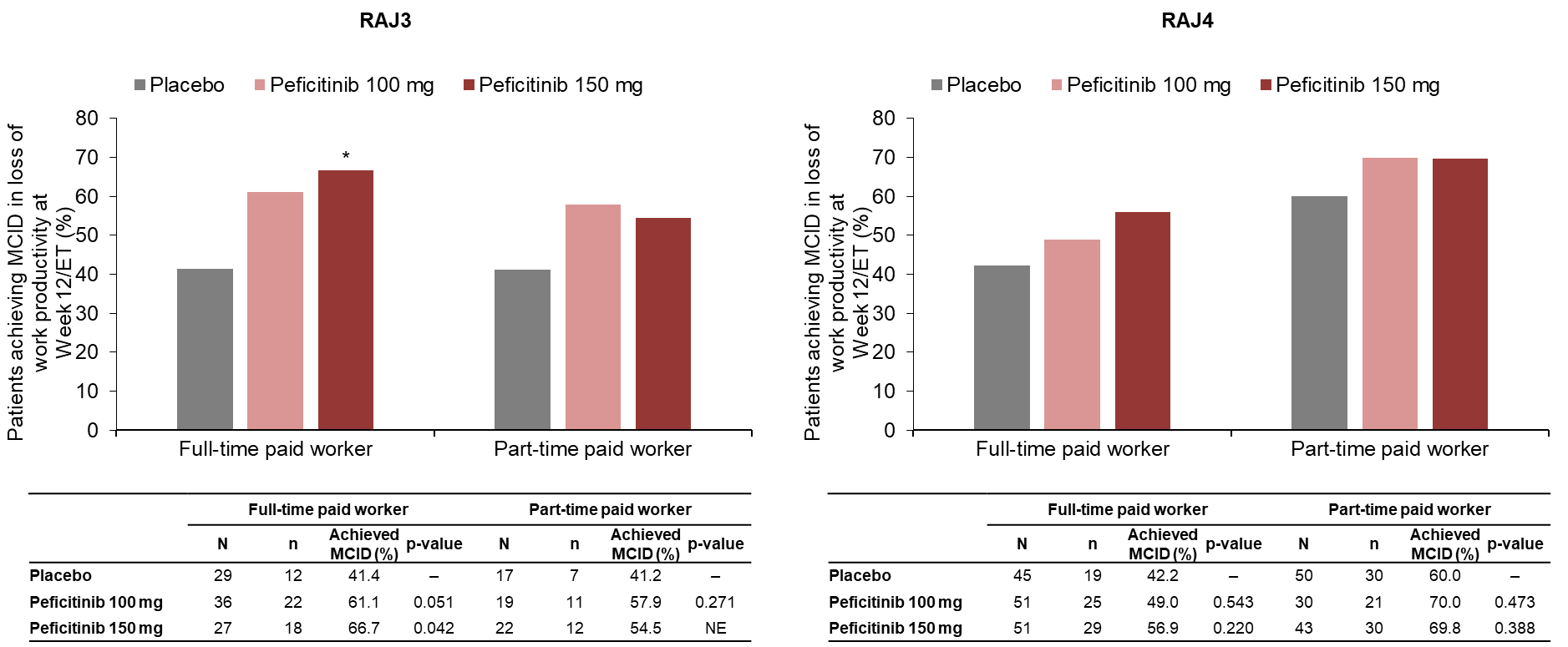


**(d)** Daily activity impairment


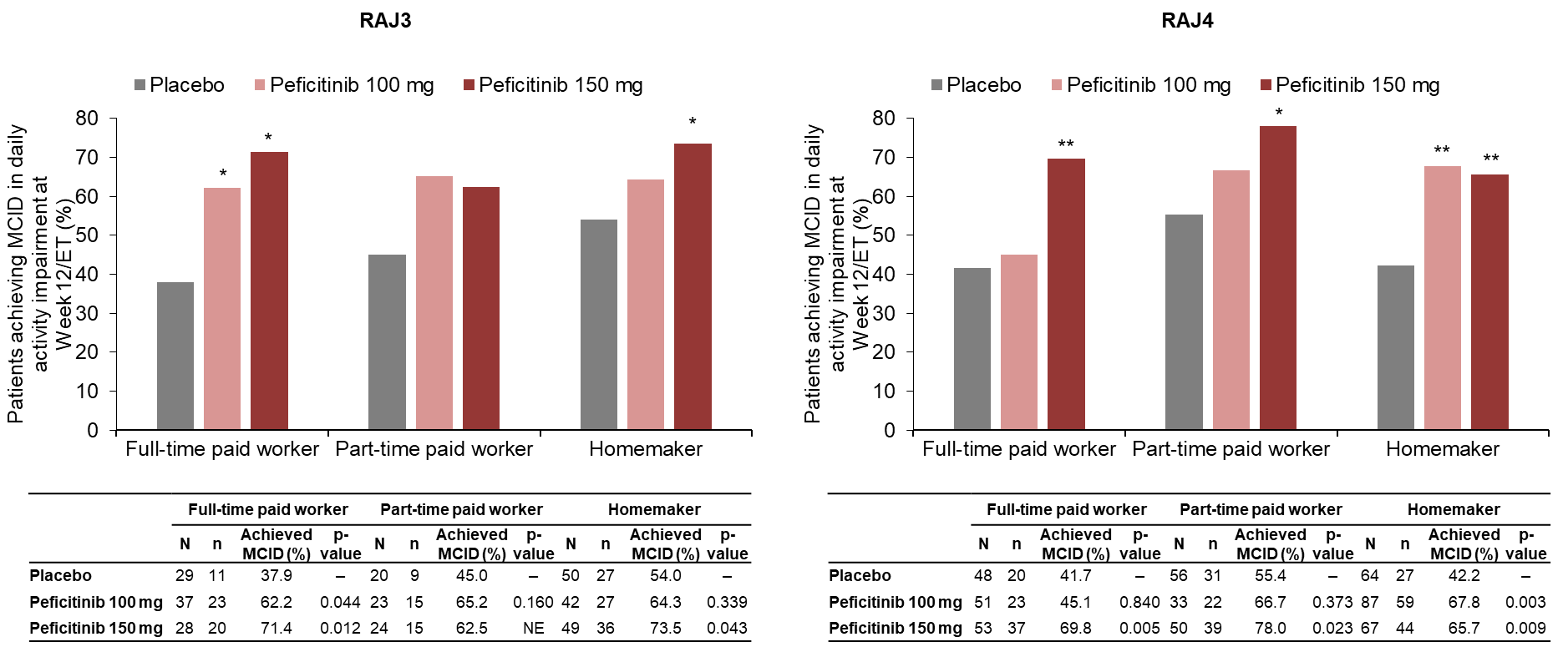

Supplement: Supplementary file 1 — Additional file 1: Supplementary Methods. Supplementary Figure 1. Correlations between WPAI domain scores and CDAI at Week 12/ET. Except for ‘daily activity impairment’, only full-time paid workers and part-time paid workers were included (not homemakers). At Week 12/ET, last observation carried forward was used for missing WPAI scores. The Pearson (r) correlation coefficients assessed the relationships between WPAI domains (absenteeism, presenteeism, loss of work productivity, and daily impairment) and clinical response (CDAI). A 95% prediction ellipse is displayed on each plot. CDAI, Clinical Disease Activity Index. ET early termination, WPAI Work Productivity and Activity Impairment. Supplementary Figure 2. Mean changes from baseline at Week 12/ET for WPAI domains by employment status. Full-time paid workers and part-time paid workers were employed for ≥35 or <35 hours/week, respectively. Homemakers were unemployed, or employed in a capacity other than paid workers, and able to perform basic activities of daily life. Only daily activity impairment data were obtained for homemakers. Higher WPAI scores indicate greater activity impairment and less productivity. At Week 12/ET, last observation carried forward was used for missing WPAI scores. p-values calculated using analysis of covariance, with no adjustment made for multiplicity. *p<0.05; **p<0.01; ***p<0.001, peficitinib versus placebo. ET early termination, WPAI Work Productivity and Activity Impairment. Supplementary Figure 3. Proportion of patients achieving MCID in WPAI at Week 12/ET by employment status. MCID for WPAI was defined as ≥7% decrease from baseline. Full-time paid workers and part-time paid workers were employed for ≥35 or <35 hours/week, respectively. Homemakers were unemployed, or employed in a capacity other than paid workers, and able to perform basic activities of daily life. Only daily activity impairment data were obtained for homemakers. All time points except for Week 12/ET are observed [file 13075_2021_2590_MOESM1_ESM.docx]
